# Behavioral Health and Learner-Centered Outcomes in Geriatric Primary Care or Community Settings

**DOI:** 10.1093/geroni/igaa057.3139

**Published:** 2020-12-16

**Authors:** Rebecca Allen, Anne Halli-Tierney

## Abstract

This symposium presents data from interdisciplinary behavioral health training and research conducted in primary geriatrics care or community settings in the Deep South. The first paper describes mixed-method learner-centered outcomes from interprofessional education case sessions. Survey and qualitative data revealed the most important experiential learning derived from collaboration, problem solving, and learning about various disciplines’ professional roles. The second paper presents longitudinal patient cognitive outcome data from the primary care, outpatient geriatrics clinic in which most of these interprofessional learners learn. Results show that only 26.2% of patients had scores indicating cognitive functioning within normal limits; 32.6% had scores indicative of mild neurocognitive disorder, and 41.2% had scores indicative of dementia. Over 30% of patients reported clinically significant levels of depression or anxiety, and 16.5% of patients reported at least one indicator of hazardous alcohol use at their baseline assessment. The third paper demonstrates that psychological inflexibility is associated with depression and anxiety at baseline, and that symptoms of depression and anxiety do not change one year later. The fourth and final paper considers the impact of hearing loss on quality of life in community-dwelling older adults. Effect size calculations indicated that adults with hearing loss who lived in the most rural regions of Alabama, had lower reported QOL scores than their counterparts who had hearing within normal limits This symposium will show why age matters in behavioral health training. Mental Health Practice and Aging Interest Group Sponsored Symposium.

